# The Optimization of the Light-Source Spectrum Utilizing Neural Networks for Detecting Oral Lesions

**DOI:** 10.3390/jimaging9010007

**Published:** 2022-12-29

**Authors:** Kenichi Ito, Hiroshi Higashi, Ari Hietanen, Pauli Fält, Kyoko Hine, Markku Hauta-Kasari, Shigeki Nakauchi

**Affiliations:** 1 Department of Computer Science and Engineering, Toyohashi University of Technology, Toyohashi 441-8580, Japan; 2 Graduate School of Informatics, Kyoto University, Yoshidahonmachi 36-1, Sakyo-ku, Kyoto 606-8501, Japan; 3 Planmeca Oy, 00880 Helsinki, Finland; 4 School of Computing, University of Eastern Finland, 80101 Joensuu, Finland

**Keywords:** computational lighting, spectral imaging, machine learning, spectrum estimation

## Abstract

Any change in the light-source spectrum modifies the color information of an object. The spectral distribution of the light source can be optimized to enhance specific details of the obtained images; thus, using information-enhanced images is expected to improve the image recognition performance via machine vision. However, no studies have applied light spectrum optimization to reduce the training loss in modern machine vision using deep learning. Therefore, we propose a method for optimizing the light-source spectrum to reduce the training loss using neural networks. A two-class classification of one-vs-rest among the classes, including enamel as a healthy condition and dental lesions, was performed to validate the proposed method. The proposed convolutional neural network-based model, which accepts a 5 × 5 small patch image, was compared with an alternating optimization scheme using a linear-support vector machine that optimizes classification weights and lighting weights separately. Furthermore, it was compared with the proposed neural network-based algorithm, which inputs a pixel and consists of fully connected layers. The results of the five-fold cross-validation revealed that, compared to the previous method, the proposed method improved the F1-score and was superior to the models that were using the immutable standard illuminant $D_{65}$.

## 1. Introduction

### 1.1. Light Sources and Optimization

Over the years, lighting technology has evolved with the development of various light sources, such as incandescent light bulbs, gas lamps, halogen lamps, tungsten bulbs, and fluorescent lamps, and technologies allowing a near-perfect reproduction of daylight, such as the light-emitting diode (LED) and organic LED technologies. Various characteristics and criteria are used to determine the value of a light source, such as the price, energy efficiency, radiation power, exothermic properties, and time required to attain a steady state. Generally, the light-source spectrum has been considered important because it affects the visibility of objects. The irradiated object’s color changes with the spectrum of the light source’s spectrum, which affects the information perceived visually, such as the object’s visibility, impression, and discrimination of objects. Historically, the effects of light sources have been quantified using the color rendering index (CRI) [1] and the color discrimination index (CDI) [2]. The CRI represents the extent of similarity between the typical object color under a light source and that of sunlight, while CDI denotes the extent to which the illuminant allows the observer to perceive the vivid color of an object. Essentially, these parameters indicate an illuminant’s colorfulness and color discrimination capacity. Accordingly, light sources have been developed for the intended purpose by using these indicators.

Recent developments in LED technology have yielded a wide variety of light sources. LED lighting is characterized by a higher energy efficiency and longer lifetime than those of traditional lighting technologies, does not require any warm-up time, and offers a spectrum featuring various peak wavelengths and spectral broadening. By combining these spectra, various spectral power distributions (SPDs) can be achieved; thus, optimal light sources can be designed according to arbitrary criteria [3]. For example, a light source that maximizes colorfulness [4], enhances psychophysical values based on experiments [5], is suitable for the appreciation of art paintings [6], improves color discrimination [7], minimizes optical damage due to light absorption and energy consumption while ensuring the color quality of artworks to avoid degradation caused by light when exhibiting artwork [8,9], and maximize the effectiveness of the Circadian rhythm for well-being [10]. Several light sources enhance the contrast between objects for the discrimination of specific objects [11,12,13,14,15,16]. In particular, the most suitable strategy for developing an optimal light source involves generating several light sources featuring various spectra and selecting the optimal spectrum. Another method is to sequentially optimize the intensity of each SPD as the lighting parameters.

When maximizing the effects of a plurality of objective indices for light sources and optimizing the parameters of the light sources, trade-off relationships among the indices must be considered. For example, it has been reported that there is a negative correlation between color rendering indices and luminous efficacy of radiation (LER) that indicates the energy efficiency of the light [17] as well as an inverse relationship between color quality and energy efficiency [18]. To obtain favorable light sources from such a combination of contradictory light-source effects, [16,17,18] have shown that optimization maximizes the Pareto efficiency between each index using a multi-objective genetic algorithm (MOGA). Convenient light sources can be examined from sets of better light-source parameters (Pareto front set) that were obtained by the multi-objective optimization. In terms of multi-objective optimization, swarm intelligence such as particle swarm optimization (PSO) is also used [19,20], since the genetic algorithm and swarm strategy indicates several good options of parameters for the objectives.

In the case of these optimization methods, only the light-source parameters with the maximum or minimum values of the objective function are selected or optimized, without other parameters such as a machine learning model. When applying this light-source optimization to a machine learning model that uses images of illuminated scenes as the input, the weights of the light source (lighting weights) and the discrimination parameters of the machine learning model should be optimized. However, because changing the SPD modifies the color information of the input images, simultaneously optimizing these two parameters in an efficient manner is a challenging task. To solve this issue, Higashi et al. [21] proposed a method for optimizing the light source; this method maximizes the performance of machine learning through iterative optimization of the SPD and alternately training the machine learning model (hereafter referred to as alternating optimization). Although this method has been validated in terms of logistic regression and linear classification using a linear support vector model (SVM), it has not been applied to more intricate and nonlinear regression or discrimination models such as deep neural networks.

Recent developments in image recognition using deep learning techniques have been remarkable. These image recognition systems can perform outstanding tasks such as general object recognition and annotation, which are difficult to achieve using conventional machine learning models. These systems can now perform more complex tasks, such as classifying images and annotating them with high precision. They provide object recognition for detecting objects in images, image segmentation that provides pixel-by-pixel classification, and valuable information, such as the posture, skeletal structure, direction of movement, estimated price, and brand of goods [22]. Light-source optimization can be applied to accelerate deep learning-based image recognition owing to its potential. Furthermore, extending the optimization method of light-source spectra to enhance machine learning performance will expand the practicality of spectral optimization and can further improve the machine vision performance.

### 1.2. Periodontal Disease

A system that modulates color information using light-source optimization can be implemented with a relatively inexpensive system consisting of a red–green–blue (RGB) camera and multiple LEDs. Using light-source optimization can reduce the cost of diagnostic assistance systems in dentistry where hyperspectral and X-ray technologies with expensive equipment are used.

Periodontal disease degrades the quality of life and affects daily life regarding pain, oral function, and aesthetics [23,24]. For example, periodontal disease may cause disadvantageous outcomes such as expensive treatment, loss of bite and chewing function owing to replacement with dentures or post crowns, worsening halitosis, and loss of appearance. Additionally, periodontal disease has been suggested to be associated with health status and other diseases such as cardiovascular and respiratory diseases [25,26]. From this perspective, maintaining proper oral hygiene and preventing worsening periodontal disease is essential for optimal quality of life and health.

In addition to visual examinations conducted by dentists, periodontal disease is diagnosed using radiographic imaging and periodontal probing to determine the progress of the periodontal disease. Generally, diagnosis via periodontal probing causes severe pain to the examinee because the probe is inserted deep into the periodontal pocket. This problem is exacerbated in low- and middle-income countries where expensive radiographic instruments are not widely available. Automatic diagnostic methods for periodontal disease via machine learning have been implemented in specific research using X-ray and hyperspectral images as the inputs [27,28,29,30]. However, both of these methods are not practical compared to diagnosis by a medical practitioner using X-rays because of the high cost of imaging and the protracted processes involved. By optimizing the light source’s SPD and changing the objects’ visibility in the visible region, color information can be enhanced in images captured by RGB cameras, which are more cost-effective than X-ray imaging equipment.

### 1.3. Spectral Imaging for Machine Learning

In most cases, spectral data are highly multivariate with collinearity compared with those measured by RGB and infrared cameras since they have many spectral channels where the spectral intensities next to each other in wavelength are similar. A typical method for dimensionality reduction of spectral data is principal component analysis (PCA). PCA finds new coordinates that transform the correlated multi-dimensional features into a new feature space where they are uncorrelated with each other, which allows dimension reduction while preserving information [31]. Also, partial least square (PLS) is used for spectral data with many variables [32]. It allows for selecting preprocessing methods and variables for regression and discrimination while avoiding the problem of collinearity between variables [33,34]. PLS is effective for analyzing spectral signals, but it is not a technique specialized for image recognition that inputs multiple pixels utilizing spatial information.

There are many examples of image recognition by deep leaning using spectral images. Researchers in [35] utilized balanced local discriminant embedding (BLDE) that linearly transforms the spectral features to reduce spectral dimensions while maximizing the margin between classes. The spatial shape information is extracted separately; the principal components of spectral images by dimensionality reduction using PCA are fed into the convolutional neural network (CNN), combined with the linearly transformed spectral information by BLDE, and used for discrimination.

Other studies in [36] proposed a recurrent model for spectral–spatial information extraction that learns the spectral information by making the spectral domain correspond to the time step and treating the spectral information grouped by pixel with a long short-term memory (LSTM). An LSTM is expected to be superior in learning relationships among spectral domains since it learns relationships among the contiguous data as conceptual information. In [37], a 3D convolution was performed to convolve the spectral and spatial domains using a 3D kernel to extract spectral information and spatial information between pixels simultaneously. Instead of increasing the number of weights by a 3D convolution, the possibility of overlearning and a vanishing gradient is suppressed by using the residual for each convolution block.

The self-attention mechanism dynamically identifies where to focus attention on the data; therefore, estimating which spatial regions and channels to focus on for learning and inference. In [38] the researchers utilized spectral attention as a spectral channel of interest, which was computed using a recurrent neural network (RNN) that mapped the spectral domain for each pixel to a time step. In [39] a module was proposed that calculated a vector of the spectral channel lengths used for spectral–spatial attention through pooling and a multi-layer perceptron (MLP) to reduce the spatial information of spectral images. 

Spectral images are used in many machine learning applications because they contain considerable information that would otherwise be compressed if imaged by a camera such as an RGB or infrared. They contribute to a high discrimination performance, especially in complex machine visions, such as deep learning. However, acquiring spectral information in the real world requires expensive equipment such as spectral cameras, spectral band-pass filters, and time-consuming photography. Thus, effective imaging systems are needed to utilize the potential of spectral information for applications of machine learning problems in the real world.

### 1.4. The Aim and Contribution of This Study

This study proposes a method for optimizing the light-source spectrum using the learning mechanism of neural networks. To date, simple RGB, infrared, and distance information have been used for image processing, and the increase in visual information using light sources has not been the mainstream approach. The increased color information by changing and optimizing the light-source spectrum may enhance the performance of machine vision systems that use images as cues. As deep learning can be applied to accomplish various tasks, if the performance can be improved by optimizing the light-source spectrum using neural networks, further advancement of machine vision technology can be rendered possible.

To confirm the effectiveness of the proposed method, we applied it to solve problems encountered in oral lesion detection using dental images to verify the optimization effect. Moreover, we compared it with the conventional method, alternating optimization, as a method for light-source optimization. Subsequently, to confirm the effectiveness of the proposed method, we applied it to lesion detection challenges using dental images to verify the effectiveness of the optimization and compared it with the conventional method, alternating optimization. Compared with the conventional method, the proposed method using a neural network can perform more complex identification with nonlinearity, and computation via the CNN can be efficient for image processing.

This study aimed to develop an optimization method for the light-source spectrum using a neural network capable of machine vision tasks. Furthermore, this method was evaluated for diagnosing periodontal disease and oral hygiene using RGB images illuminated by an optimal light source.

Since the proposed system uses images captured via RGB or infrared cameras under multiple light sources while utilizing spectral information, our system can be realized at a lower cost in terms of time and price than machine learning systems that use features captured by the spectral imaging system. In addition, the proposed system is expected to contribute to the spread of low-cost and high-accuracy machine vision systems.

## 2. Materials and Methods

### 2.1. The Color Observation Model

**Figure 1:**
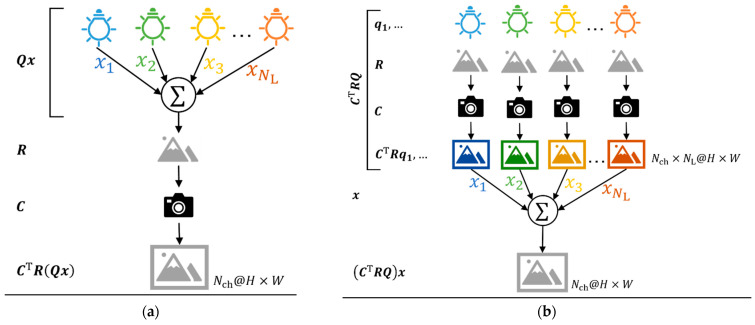
The observed features: (**a**) The image was measured under the accumulated (mixed) spectral power distribution (SPD) of sub-SPDs with the intensities of $x=\left[x_{1},\;\ldots ,x_{N_{L}}\right]$. (**b**) Images were measured for each sub-SPD, then synthesized into one image by multiplication with the linear weights, x. Equivalence was achieved between the case observed under the accumulated spectral power distribution (SPD) and a linear synthesized variant of the observed features under each sub-SPD. Considering that the observed features that were measured under the accumulated SPD ($=\left(C^{T}RQ\right)x$ ) and the synthesized features, after measuring under the sub-SPDs ($=C^{T}R\left(Qx\right)$ ), are the same, from Equation (3), the images in (**a**,**b**) are the same if lighting weights, x, are the same as each other and measuring the images by camera has linearity.

Assuming a machine learning model that performs identification and regression based on the features of the subject under a light source, the features are defined according to the following color observation model. When $N_{L}$ light sources featuring different spectral intensity distributions (hereafter referred to as sub-light sources) simultaneously illuminate at their respective intensities, the spectral intensity distribution $l\left(\lambda \right)$ of the light sources can be expressed as follows:

(1) $$l\left(\lambda \right)=\overset{N_{L}}{\underset{i}{\sum }}x_{i}q_{i}\left(\lambda \right),$$

where $q_{i}\left(\lambda \right)$ denotes the SPD of the i-th sub-light source ($i=1,2,\ldots ,N_{L}$), and $x_{i}$ is the weight of intensity of each sub-light source. When observing an object illuminated by a light source, the observed feature $o_{j}$ of the j-th channel of the object is expressed as:

(2) $$o_{j}=\int c_{j}\left(\lambda \right)l\left(\lambda \right)r\left(\lambda \right)d\lambda ,$$

where $c_{j}\left(\lambda \right)$ represents the sensitivity distribution of the j-th channel sensor of the observer (e.g., camera or human eye), $l\left(\lambda \right)$ symbolizes the SPD of the light source, and $r\left(\lambda \right)$ depicts the reflectance of the object. When observing a transparent object, $r\left(\lambda \right)$ denotes the transmittance:

(3) $$\begin{array}{l} O=C^{T}RQx\\ \;=Ax\;\left(∵A=C^{T}RQx\right),\; \end{array}$$

where $C\in {\mathbb{R}}^{N_{\lambda }\times N_{\mathrm{ch}}}$ represents the matrix summarizing the spectral response of channel i shown as $c_{i}\in {\mathbb{R}}^{N_{\lambda }}$ and $C=\left[c_{1},\;\ldots ,\;c_{N_{\mathrm{ch}}}\right]$, $R\in {\mathbb{R}}^{N_{\lambda }\times N_{\lambda }}$ denotes the reflectance matrix with its diagonal component representing the reflectance to be observed and the other is zero, $Q\in {\mathbb{R}}^{N_{\lambda }\times N_{L}}$ denotes a matrix summarizing the SPD of the i-th sub-light source as $Q=\left[q_{1},\ldots ,\;q_{N_{L}}\right]$, and $x\in {\mathbb{R}}^{N_{L}}$ symbolizes the weight of intensity as $x={\left[x_{1},\ldots ,\;x_{N_{L}}\right]}^{T}$. Equation (3) indicates that the feature O is equal when the features photographed under the sub-light sources are added with the lighting weights, x, and when the features are photographed under the spectrum composed of the lighting weights, x (Figure 1).

Upon determining the sensitivity of the camera and the spectra of the sub-lights, simply photographing the subject under each sub-light will yield A before optimization, thus eliminating the need for repeated spectral optimization and photography, as demonstrated by Liu et al. [13]. Additionally, compared with methods that use spectral images captured using diffraction grating or narrow-pass filters, the use of simple RGB or infrared cameras is expected to reduce the requirements in terms of time and price.

### 2.2. Alternating Optimization: A Light-Source Spectrum Optimization for Machine Learning

Alternating optimization is an innovative method for optimizing the light-source spectrum, aiming to maximize the performance of machine learning [21]. The method proposed in this study is based on alternating optimization to advance the application of light-source spectrum optimization to machine vision. This section presents the details of alternating optimization, which forms the basis of the proposed method.

We introduce the concept of alternating optimization, which is a conventional method of optimizing the light-source spectrum, while minimizing the performance loss of machine learning models [21] using a linear discriminant model that infers y based on the observed sensor value, O, as expressed in Equation (3). The infrared value $\hat{y}$ can be formulated as:

(4) $$\begin{array}{l} \hat{y}=f\left(w^{T}O+b\right)\\ \;=f\left(w^{T}Ax+b\right), \end{array}$$

where $w\in {\mathbb{R}}^{N_{\mathrm{ch}}}$ denotes the weights of the linear model, $f\left(\cdot \right)$ is a function, such as the logistic sigmoid function transforms the input to range $\left[-1,\;1\right]$ because target class $y\in \left\{-1,\;1\right\}$ for classification tasks, and b is a bias for boundary. In this method, to optimize the linear discrimination expressed in Equation (4), A is used as the training sample, and the discriminant weights, w, and lighting weights x are treated as the parameters to be trained. These weights are optimized by repeating the optimizations presented in Equations (5) and (6):

(5) $$w=\arg\underset{w}{\min}J\left(w|x\right),$$

(6) $$x=\arg\underset{x}{\min}J(x|w),$$

where $J\left(\cdot \right)$ symbolizes the cost function of the discriminant model. In the case of discrimination with the SMV, the model requires only a vector of linear weights and kernel parameters to determine multiple separating hyperplanes, so the solution is obtained immediately. By dividing the optimization of w and x into their subproblems, the space of parameters to be optimized is less complex than when w and x are optimized simultaneously, and the solution can be obtained stably in the alternating optimization.

When applying this alternating optimization to complex machine learning models such as deep neural networks (DNNs), a large number of weights to be optimized must be considered. Although discriminative hyperplanes represented by a small number of parameters, as in the case of SVM, are relatively simple, deep learning models such as DNN have many discriminative weights. In a complex hyperspace with many parameters, there are many local optimums, and the possibility of getting caught in them is higher, making it more challenging to arrive at a better solution.

If the NN model’s weights $W_{\mathrm{NN}}$ are trained in lighting weight x until the cost $J(W_{\mathrm{NN}}|x)$ converges, thus, many parameters have fallen into their local optimum. So even if x is subsequently optimized via cost $J\left(x|W_{\mathrm{NN}}\right)$, it will be hard to change dynamically to remove it from the local optimum. Therefore, when training a neural network in the alternating optimization, we face the practical problem: how many epochs is efficient to switch over. Also, if the number of parameters of x is small compared to $W_{\mathrm{NN}}$, the difference between the subproblem $\underset{W_{\mathrm{NN}}}{\min}J(W_{\mathrm{NN}}|x)$ and the whole problem $\underset{W_{\mathrm{NN}},\;x}{\min}J\left(W_{\mathrm{NN}},\;x\right)$ will be small. Thus, there will be little merit in dividing the problem into subproblems and optimizing them alternately. In other words, there are better options than alternating optimization for lighting optimization for machine leazcrning models with complex parameters such as NN.

Therefore, in the next section, we show a method to optimize both the NN model and the lighting weight x simultaneously through backpropagation by incorporating x into the NN model. Since the gradients are computed in a backpropagation process for $\underset{W_{NN}}{\min}J\left(W_{NN}|x\right)$ and $\underset{W_{NN},\;x}{\min}J\left(W_{NN},\;x\right)$ in either case, the proposed method that optimizes x simultaneously is more efficient.

### 2.3. The Optimization of the Light-Source Spectrum Using a Neural Network

**Figure 2:**
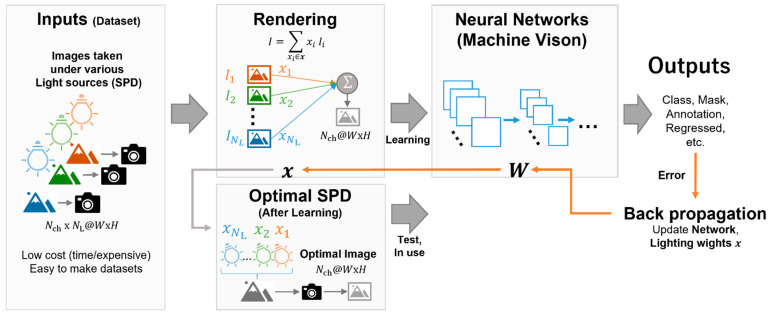
The concept of optimization of the light-source spectra using a neural network (NN). The input to the model is a set of images of objects taken with the same camera under sub-spectral power distributions (SPDs) that have different spectral proportions from each other, where each sample is composed of $N_{L}$ number of sub-light images of $N_{\mathrm{ch}}$-channels. The input image is linearly combined according to the lighting weights x to obtain a single image with $N_{\mathrm{ch}}$-channels (rendering process), fed into the NN model. During training, the NN model’s weights W and x are updated simultaneously according to the gradients, which are computed by backpropagation from the cost (error) of the NN model’s output. After sufficient training, the accumulated SPD obtained by mixing the sub-SPDs with intensities of x becomes the optimal light source for the machine learning problem. The image measured under the optimal light source is equivalent to the image obtained by the rendering process. This can be entered into the model for inference with fewer shots of images than when the dataset was obtained.

Inspired by alternating optimization, we propose a model that optimizes and discriminates between light sources using a neural network. The model consists of a light-source synthesis (rendering) component and a neural network model (Figure 2). It accepts the input, $A\in {\mathbb{R}}^{N_{\mathrm{ch}}\times N_{L}}$, which is expressed in Equation (3), and subsequently renders the RGB image, which is calculated using the weighted sum of the sub-light components of the A-matrix with the lighting weight, x. This weight, ***x***, is used for rendering, and the optimized light source is reproduced using the weighted sum of the SPD of the sub-light with x. The infrared value $\hat{y}$ can be represented as:

(7) $$\begin{array}{c} \hat{y}=f\left(f_{\mathrm{NN}}\left(A|x,\;W\right)\right)\\ =f\left(f_{\mathrm{NN}}^{\prime }\left(Ax|W\right)\right), \end{array}$$

where, $f_{\mathrm{NN}}\left(\cdot \right)$ denotes the computation of the neural network containing the rendering process, $f_{\mathrm{NN}}^{\prime }\left(\cdot \right)$ denotes the neural network without rendering, W are the weights of the neural network, and $f\left(\cdot \right)$ denotes an activation function of the output layer. Thereafter, the rendered image is used as the input for the consequent neural network models, and the image processing is performed based on the model. This model can be replaced with a conventional model that flexibly utilizes the rendered image. Therefore, this model is feasible for other tasks such as image classification, masking, and annotation.

**Figure 3:**
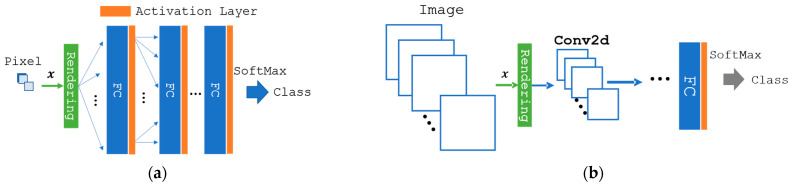
A classification model featuring the optimization of light-source spectra: (**a**) the model with only a fully connected layer (FCL), (**b**) the model featuring an FCL and a convolutional neural network (CNN). In the rendering layer, input images are linearly combined with lighting weights x**.** Rendered images are fed into the fully connected layer (FC) in (**a**). The referenced class of the model is calculated as a one-hot coding with the softmax of FC’s output. In the case of (**b**), the rendered image is fed into a few 2D-convolution layers (Conv2d) and then into an FC. Eventually, the class is referenced via softmax, the same as (**a**).

The features under the optimal light source composed of the sub-light sources can be computed using a weighted sum of the lighting weights of the features under these sources (Figure 1). As the output to the subsequent layer is the sum of the product of the lighting weights, x, and the input, it is differential by x. Suppose the light-source optimization is applied to the neural network model that is end-to-end; in this case, every layer’s outputs in the model are differentiable by the weights, thereby rendering it an end-to-end learning model. A neural network with a fully connected layer, convolutional layer, and fully connected layer (FCL) was applied to the model to validate the proposed optimization method (Figure 3).

### 2.4. Problem Setting for Oral Lesions Detection

To verify the effectiveness of the proposed method, we focused on the problem encountered in classifying oral lesions. Herein, we present the problem scenario. Table 1 summarizes the problem settings.

#### 2.4.1. The One-vs-Rest Classification

The one-vs-rest classification was used to classify the classes of the input image using 1-pixel or 5×5 patch images for the healthy enamel portion of the tooth and the attrition and erosion, calculus, initial caries, microfracture, and root lesions, respectively. 

#### 2.4.2. The Input and Output

As the input, we used RGB 3-channel images calculated using Equation (3), 1×1 pixels for alternating optimization and NN-based source-spectrum optimization, and 5×5 patch images for the CNN-based source-spectrum optimization.

**Figure 4:**
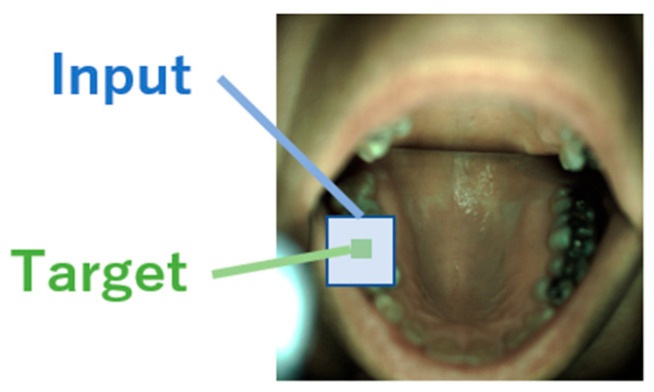
A sample of the target class for 5×5 patch images, adapted from the image of ODSI-DB [40] (CC BY-NC-SA 4.0).

The output for a 1×1 pixel is the class of that pixel. Only the class of the central pixel of the image was used as the output for samples of 5×5 patch images (Figure 4).

#### 2.4.3. Initial Lighting Weights

Three types of initial vectors were used to verify the difference in the initial vectors of the lighting weights:

- A randomized vector in the range of $\left[-1,\;1\right]$;

- Weights which construct a cumulative SPD using weighted sub-lights that approximates the D65;
- Uniform weights with **1**: $1^{N_{L}}=\left[1,\;1,\;\ldots ,\;1\right]$.

Randomized initial vectors were used for optimization by utilizing the 400–830 nm band, and other initializations were used only for the CNN-based optimization by utilizing the 400–1000 nm band. The approximation of D65 consisting of sub-lights was formulated based on the following assumption:

(8) $$q_{D65}=Qx,$$

where $q_{D65}\in {\mathbb{R}}^{N_{s}}$ denotes the vector of the SPD, **N_s_** is the number of spectral components, and the lighting weights for the sub-lights are:

(9) $$x_{\mathrm{approx}}=Q^{+}q_{D65},$$

where $Q^{+}$ represents the pseudo-inverse of the SPD matrix of sub-light $Q\in {\mathbb{R}}^{N_{s}\times N_{L}}$, x denotes the estimated lighting weight of the sub-light to approximate the SPD of D65. Consequently, the approximated distribution, $q_{D65}^{\prime }$, is the product of the sub-lights and the weights $x_{\mathrm{approx}}$:

(10) $$q_{D65}^{\prime }=Qx_{\mathrm{approx}}.$$

#### 2.4.4. The Cost Function for Alternating Optimization

A linear SVM was used for each optimization in the alternating optimizations shown in Section 2.2. SVM solves the decision hyperplane to maximize margins from samples. The margin, which is the distance between hyperplanes, determines the decision boundary from a sample A from Equation (4) is formulated as:

(11) $$d\left(w|A,x\right)=\frac{\left|w^{T}Ax+b\right|}{||w||},$$

(12) $$d\left(x|A,w\right)=\frac{\left|w^{T}Ax+b\right|}{||x||},$$

where b is a bias of the hyperplane. To make a decision for the sample means keeping:

(13) $$y_{i}\left\{w^{T}A_{i}x+b\right\}\geq 1,$$

where $i=1,\ldots N_{s}$, $N_{s}$ is number of samples, and $y_{i}\in \left\{-1,\;1\right\}$ is for target of the decision for the i-th sample. Equation (13) holds equal for the nearest sample and the margin are $d\left(w|A,x\right)=\frac{1}{||w||}$ and $d\left(x|A,w\right)=\frac{1}{||x||}$**,** respectively. Thus, the cost function corresponding to the expressions in Equations (5) and (6) is the inverse of the margin and the optimization problems, as follows:

(14) $$\begin{array}{l} \underset{w}{\min}\;J(w|x),\\ \mathrm{where}\;J(w|x)=||w||,\\ {\mathrm{subject}\;\mathrm{to}\;y}_{i}\left\{w^{T}A_{i}x+b\right\}\geq 1,\;i=1,\ldots ,N_{s}. \end{array}$$

(15) $$\begin{array}{l} \underset{x}{\min}J(x|w),\\ \mathrm{where}\;J(x|w)=||x||,\\ {\mathrm{subject}\;\mathrm{to}\;y}_{i}\left\{w^{T}A_{i}x+b\right\}\geq 1,\;i=1,\ldots ,N_{s}. \end{array}$$

#### 2.4.5. The Cost Function for the Neural Network-Based Optimization

The softmax function was used for the activation function of the output layer in Equation (7) for the multi-class classification and cross-entropy loss expressed in Equation (16), which was used for the cost function in the NN-based and CNN-based optimization models, as follows:

(16) $$J\left(\hat{y},\;y\right)=-\overset{C}{\underset{c=1}{\sum }}\log\left(\frac{\exp\left({\hat{y}}_{c}\right)}{\sum_{j=1}^{C}\exp\left({\hat{y}}_{j}\right)}\right)y_{c},\;$$

where $\hat{y}=\left[{\hat{y}}_{1},\ldots ,{\hat{y}}_{C}\right]$ with ${\hat{y}}_{j}$ from $\left[0,\;1\right]$ symbolizes the vector of the score of class j, y depicts a one-hot vector, $\left\{v\in {\left\{0,1\right\}}^{C}|\;\overset{C}{\underset{i=1}{\sum }}v_{i}=1\right\}$, for the truth class, and C represents the number of classes.

#### 2.4.6. The Grid Search and Trials

A grid search was performed to find better hyperparameters for the NN and CNN-based models, since the NN-based models have many hyperparameters which determine the model’s behavior and means of learning, such as the number of layers and weights for each layer, type of activation function, and learning rate. Parameters tried in the grid search are listed in Table 2. 

The alternating optimization was conducted ten times and different initial weights were used in each trial.

### 2.5. The Materials for Oral Lesions’ Detection Problems

The periodontal spectral images, SPDs of sub-light sources, and the sensitivity distribution of the RGB camera as an observer’s sensor were used to simulate oral images under lights with an arbitrary SPD. The corresponding details are presented in this section.

#### 2.5.1. Light Sources

**Figure 5:**
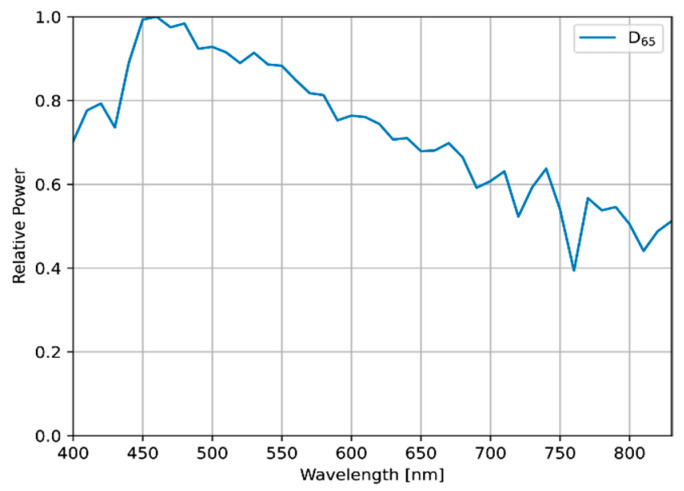
The SPD of D65, which was used as the reference light source.

For comparison with optimized lights, the standard illuminant D65 (defined by the International Commission on Illumination) was used as a reference light source (Figure 5), and two types of SPD were used as sub-lights to reproduce various spectra for optimization, as follows:

- Measured SPDs of 24 real LEDs in the 400–830 nm band;
- Simulated SPDs, with their mean aligned at even intervals, in the 400–1000 nm band.

**Figure 6:**
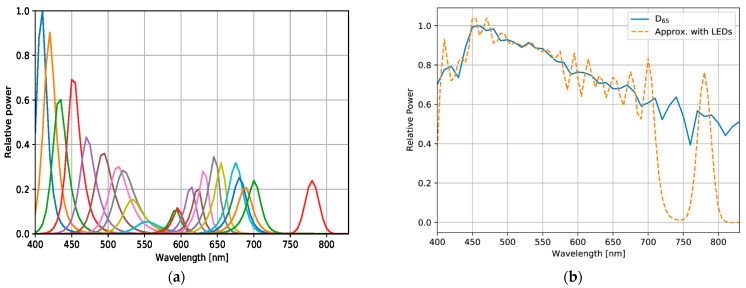
Sets of SPDs with real light-emitting diodes (LEDs): (**a**) measured SPD of 24 LEDs for sub-lights, and (**b**) approximation of an SPD of D65 standard illumination with SPDs represented in (**a**).

**Figure 7:**
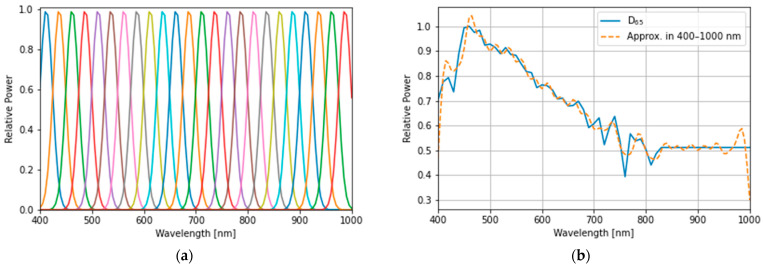
(**a**) The simulated sub-light source: each spectral distribution was computed by assuming a Gaussian distribution with a full-width at half maximum (FWHM) wavelength, $\lambda_{\mathrm{FWHM}}=40\;\mathrm{nm}$, and its means are 412, 437, 462, 487, 512, 537, 562, 587, 612, 637, 662, 687, 712, 737, 762, 787, 812, 837, 862, 887, 912, 937, 962, and 987 nm. (**b**) The approximation of the D65 standard illumination spectral distribution with band-adjusted sub-light sources is represented in (**a**).

These SPDs have different peaks and proportions (Figure 6 and Figure 7, and Table 3). The approximated SPD corresponds to the 24-LEDs sub-lights, and compared to D65, the wavelength ranges covered by the sub-lights are 720–760 and 800–830 nm. This result indicates that the sub-light can be disadvantageous compared to D65, and thus, the spectral information in the lacking bands cannot be utilized. For a fair comparison with the reference, the wavelength range covered by the sub-lights should be aligned with D65. The distribution of the D65 light source can be reproduced by combining it with a sub-light SPD that has been adjusted to cover the entire wavelength range. With the simulated sub-lights, the optimized SPD may have a similar distribution to D65 and result in a similar score, or it may be more optimal compared with D65, resulting in a higher-performing light source.

#### 2.5.2. The RGB Camera

**Figure 8:**
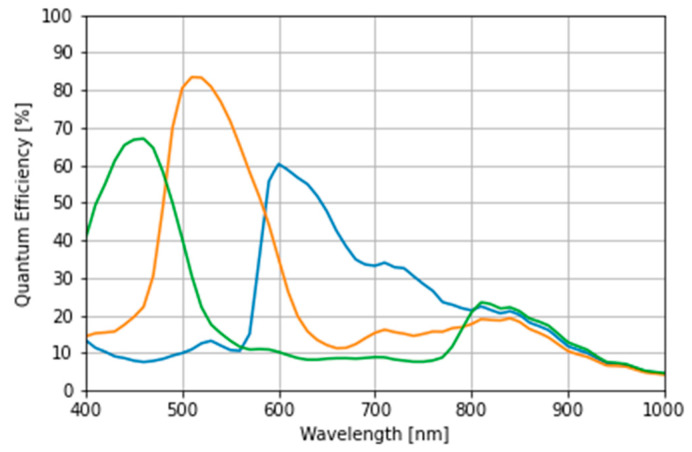
The quantum efficiency of complementary metal oxide semiconductor-based red–green–blue camera: AR1335.

To simulate the observation of the illuminated object, the spectral sensitivity of an RGB camera (AR1335; ON Semiconductor, Phoenix, AZ, USA) was used as the image sensor (Figure 8).

#### 2.5.3. Datasets

**Figure 9:**
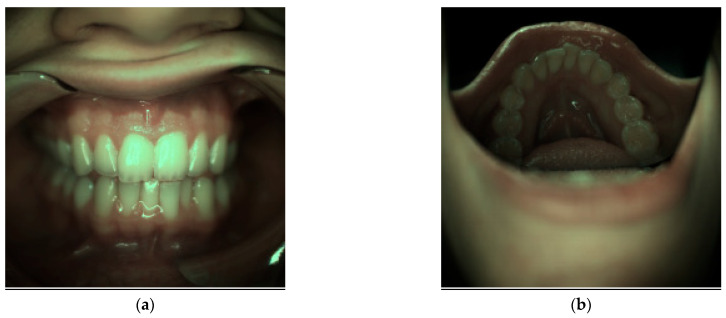
Samples of the periodontal image from ODSI-DB [40] (CC BY-NC-SA 4.0). (**a**,**b**) are rendered from reflectance spectral image computationally with camera sensitivity in Figure 8 and spectral power distribution of D65 in Figure 5.

The reflectance spectral images were obtained from the oral and dental spectral image database dataset [40]. The Computational Spectral Imaging Laboratory published these images at the University of Eastern Finland, Joensuu campus (Joensuu, Finland), and the Institute of Dentistry at the University of Eastern Finland Kuopio campus (Kuopio, Finland). Additionally, annotations of 40 classes as ground truths were obtained from a dental expert’s assessment. Figure 9 shows a sample of the periodontal image under the D65 from the dataset.

To validate the proposed optimization method, the two-class classification problem of one-vs-rest was assumed to distinguish the target class and the other in each lesion on teeth and enamel as a healthy part of teeth. In this study, we focused on five lesions on the teeth: attrition/erosion, calculus, initial caries, and microfractures. For comparison of the methods, 1×1 pixels for alternating optimization, the proposed method with fully connected NN and the 5×5 patch images for CNN were selected (Table 4). The sample size for each class in the one-vs-rest classification problem is presented in Table 5.

The dataset contains spectral reflectance images obtained via two types of cameras with different spectral resolutions: 450–950 nm with 10-nm band steps, and 400–1000 nm with approximately 3-nm band steps. From the dataset, images containing the 400–830 nm band were selected and subsequently interpolated for the wavelength axis into a 5-nm band step resolution.

## 3. Results and Discussion

To verify the proposed method, a classification was performed for each method under the conditions listed in Table 2. A five-fold cross-validation was conducted to measure the scores for alternating optimization and optimization using neural networks. As indicated in the results of the alternating optimization, the cost function of the optimization converged in all trials, and the best score in all trials was subsequently selected based on the F1-score. In the methods utilizing NN and CNN, a grid search was conducted using sets of hyperparameters, as detailed in Section 3.1. The optimal models for each classification problem were selected based on the F1-score of the cross-validation among the models with sets of parameters in the grid search.

### 3.1. The Performance Comparison among Methods

**Figure 10:**
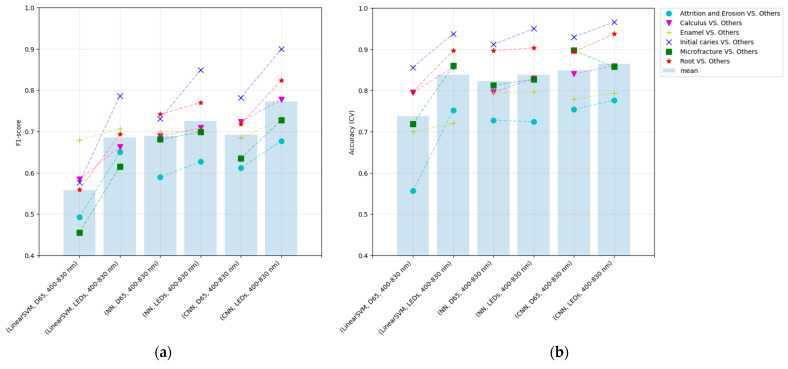
The performance of each method in the five-fold cross-validation: alternating optimization (linear-SVM), the proposed method with only FCL (NN), and the proposed method with CNN (CNN): (**a**) F1-score and (**b**) accuracy.

**Figure 11:**
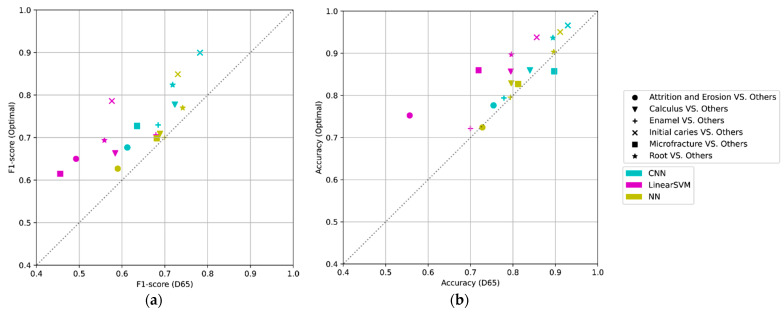
The comparison of performance between the reference (D65) vs. the optimal light in the five-fold cross-validation: alternating optimization (linear SVM), proposed method with only FCL (NN), and proposed method with CNN (CNN): (**a**) F1-score and (**b**) accuracy.

As a learning result, the classification F1-score of the NN and CNN on the optimal light source exceeds that of the previous method in the average of each class (Figure 10). Among all methods, the optimization with CNN yielded the best performance results. In all optimizations, the F1-score is greater than that in the reference light (D65) case (Figure 11), indicating that the optimization method involving the light-source spectrum improved the performance of the classification. 

Comparing the results of the NN and CNN with the D65 light source revealed that the average F1-score is approximately identical, suggesting that the CNN does not effectively employ peripheral information in the image input by the D65. In contrast, the performance of the CNN with the optimal light source was improved compared to the D65, suggesting that the enhancement of input information by optimizing the light-source spectrum may be effective in more complex feature spaces. The optimization model with CNN accepts the 5×5 image that contains information regarding the area surrounding the pixel of interest and possesses a more complex feature space than as the 1×1 input that does not possess this information.

### 3.2. The Effect of NIR Information and Specific Initial Lighting Weights

**Figure 12:**
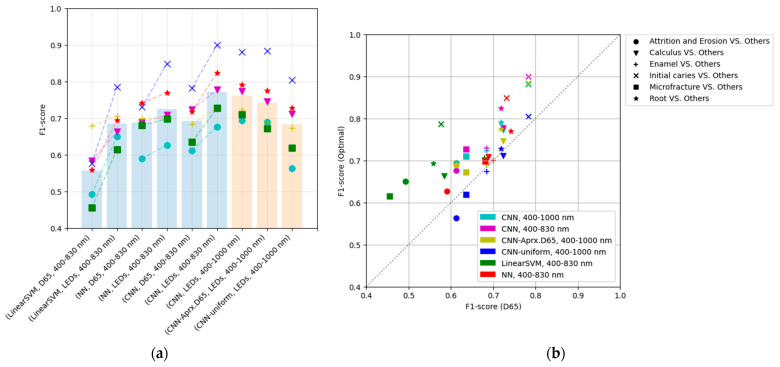
The performance of each method of the band-aligned SPD utilizing the NIR in the 400–1000 nm band with the proposed method with CNN: (**a**) F1-score for each method, and (**b**) comparison of performance between the reference (D65) and the optimal light. In the three models utilizing the 400–1000 nm band, the lighting weights were initialized in different ways: randomized in [0,1], approximating D65, and as uniform vector 1, respectively.

A grid search for the CNN-based optimization was conducted using band-adjusted sub-lights (Figure 12). Three cases of the initial vector of lighting weights were used: randomized case, approximation of D65, and approximation of uniform weights.

In the learning results, optimization using band-adjusted sub-lights in the near-infrared (NIR) range (400–1000 nm) exhibits inferior performance compared to the results obtained using the 400–830 nm range. The following two factors are suggested to have simultaneously affected the cause of performance decline:

- The camera sensitivity in the NIR region does not differ between the three RGB channels (Figure 8) and no information appears in the RGB channel;
- Upon extending the wavelength range to 1000 nm and evenly spreading them, the variety of the cumulative SPD is lost, as represented by the sub-light in the distinct wavelength band of 400–800 nm.

The performance may be improved by using sub-lights evenly distributed over a narrower range where differences in sensitivity of RGB appear, such as 400–800 nm, or by using the four-channel information from an RGB-NIR camera with an additional channel exhibiting sensitivity in the infrared region.

Comparing the initial weights’ differences revealed that the randomized vector results offered a superior performance. In addition, the optimization result using the initial value that approximates D65 is superior to that using D65, which suggests lighting weights that are superior to D65. In a randomized manner, positive and negative weights are input as the initial values, but in approximating the D65 and uniform weights, only the positive or negative values are obtained, which is considered a disadvantage; consequently, the performance is lower than that of the randomizing method. 

A randomized initial vector yields the highest performance and is more optimal than using D65. In particular, random initialization is considered sufficient without any special effort regarding the initial lighting weights.

### 3.3. The Optimal SPD and Stability

**Figure 13:**
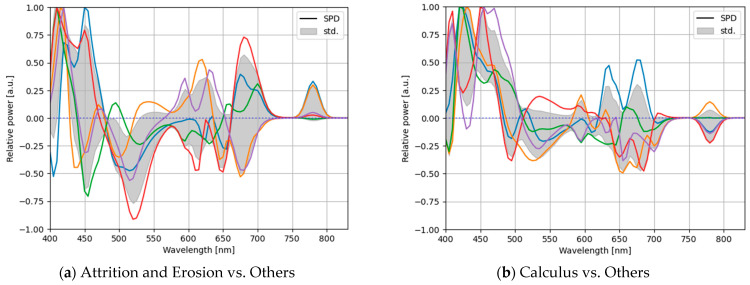

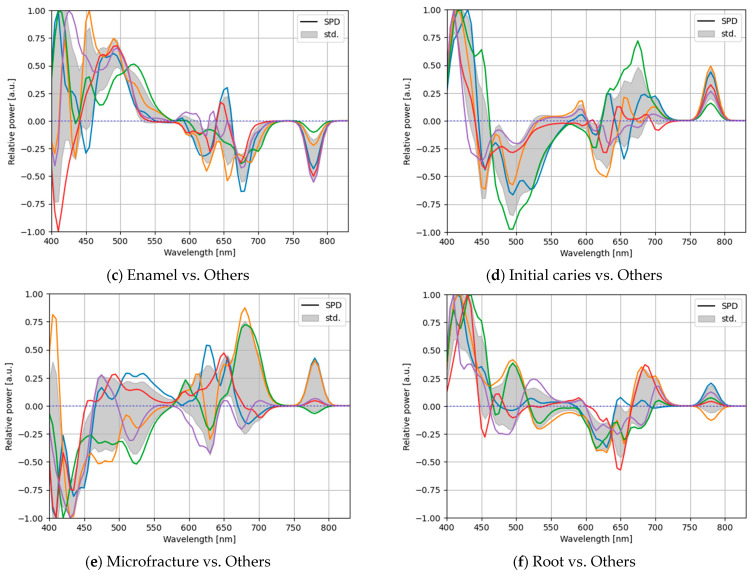
The optimal SPDs of the CNN-based optimization in a five-fold cross-validation with the measured LEDs having spectral powers in the 400–830 nm band for each classification of one-vs-rest classification: (**a**) attrition and erosion vs. others, (**b**) calculus vs. others, (**c**) enamel vs. others, (**d**) initial caries vs. others, (**e**) microfracture vs. others, and (**f**) root vs. others.

To analyze the stability of the optimization, we computed the optimized light source from the lighting weights obtained by the CNN-based optimization, which yielded the highest performance among all the methods (Figure 13).

Evidentially, the optimal light sources obtained via the five-fold cross-validation possessed different proportions and did not exhibit the same light-source weights. As different light-source weights were learned, the solution via the CNN-based light-source optimization did not yield the optimal global solution. In addition, because the solution was not proven to be at least locally optimal, it could not be considered the optimal local solution. From this result, we concluded that a better solution could be obtained, and the performance could be improved. To obtain a more optimal local solution for the light-source weights, after training the model, we can update only the lighting weights while fixing the training weights of the model, except for the lighting weights. Moreover, the lighting weights are different for each training, indicating that the optimization may not be verifiably stable. Multiple trials with different initial values under the same hyperparameters are necessary to find a better solution.

The feature space to be explored is vast and complicated because many parameters, including the training weights of the neural network, change during training. To improve the stability of the solution of the lighting weights, a pre-trained model, such as that pre-trained using the input image of a fixed light source such as D65, can be used as the training weights of the neural network. Although obtaining stable solutions for lighting weights is a challenging task owing to a large number of training parameters of the neural network and the complexity of the search space, the stability can be improved via mechanisms to avoid overfitting, such as pre-training and dropout, in neural network training.

### 3.4. The Distances between Classes in the Enhanced Feature Space

The distances between the target class and dataset samples in the feature space were analyzed to reveal how color enhancement with optimal light improved the discrimination performance. A bigger interclass distance means tolerance of the classification to noise caused by the ambient light in actual measurements. Therefore, the interclass distance is useful as a metrics of robustness.

**Figure 14:**
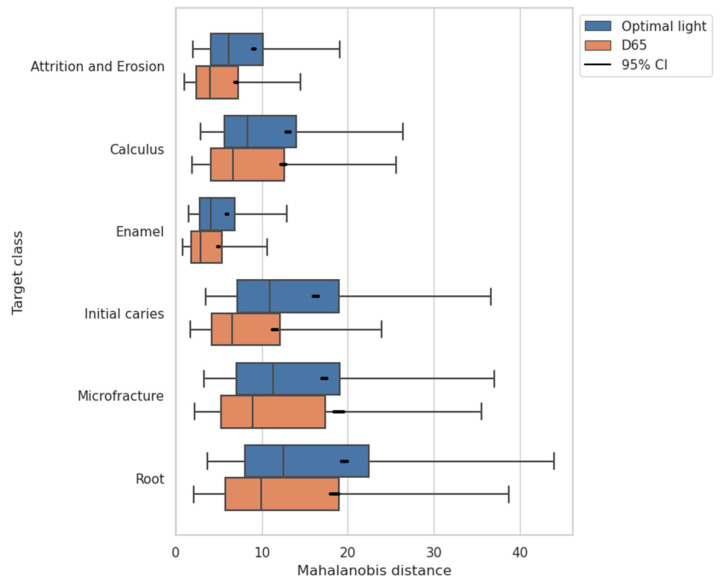
The Mahalanobis distance of dataset samples from the distribution of target class in feature space for each one-vs-rest classification. The features have 75 dimensions consisting of three channels of RGB at 5×5 patch images rendered under the D65 or under the optimal light with the CNN-based method.

The Mahalanobis distance is the statistically-defined distance between the center of the probability distribution of the class in interest and a sample in the feature space normalized by the variance–covariance matrix of the distribution of interest. In outlier detections for abnormal samples and classifications such as “healthy” or “unhealthy,” the Mahalnobis distance can be used as a scale of samples’ features [41,42]. Regarding the one-vs-rest classification, the distance of a sample’s feature x in the ‘rest’ class from the probability distribution D of the target class c as ‘one’ in one-vs-rest is computed as:

(17) $$d_{M}\left(x,\;D\left(\mu_{c},\;\Sigma_{c}\right)\right)=\sqrt{{\left(x-\mu_{c}\right)}^{T}\Sigma_{c}^{-1}\left(x-\mu_{c}\right)}$$

where $x={\left[x_{1},\ldots ,x_{N_{d}}\right]}^{T}$ is the feature vector of a sample with $N_{d}$ dimension and D is the multivariate distribution described by mean of features $\mu_{c}={\left[\mu_{c,\;i},\ldots ,\mu_{c,\;N_{d}}\right]}^{T}$ and variance-covariance matrix $\Sigma_{c}\in {\mathbb{R}}^{N_{d}\times N_{d}}$ of class c, hence $\mu_{c}$ and $\Sigma_{c}$ are calculated as:

(18) $$\mu_{c}=\frac{1}{N_{c}}\overset{N_{c}}{\underset{i}{\sum }}y_{c,\;i},$$

(19) $$\Sigma_{c}=Y_{c}^{T}Y_{c},$$

where $Y_{c}\in {\mathbb{R}}^{N_{c}\times N_{d}}$ denotes the data matrix for $N_{c}$ samples of class c, and $y_{c,\;i}\in {\mathbb{R}}^{N_{d}\times 1}$ denotes a feature vector of i-th sample of class c. The feature space has $N_{d}=75$ dimensions in the case of the CNN-based classification from a 5×5  patch image with three channels of RGB. The comparison of the Mahalanobis distances between the proposed CNN-based method and the D65 without optimization is shown in Figure 14. The distances are more significant for the result of the optimal lights with the CNN-based method than the D65.

According to the result of expanded interclass distances (Figure 14) and the improved discrimination performance (Figure 11), we can say that RGB information containing more valuable spectral information is obtained with the lighting learned through the proposed method and contributed to the more accurate classification. In addition, measurements with an optimal light source with an increased interclass distance indicated the possibility of being more resistant to noise due to ambient light than measurements under a standard light source.

## 4. Limitations and Future Work

This study performed a two-class one-vs-rest classification to verify the proposed method. In addition, considering the demand for image processing, the two-class classification task is limited, and validating the proposed method is a prerequisite to its application in further tasks, such as multiclass classification and annotation.

Although the light-source spectrum optimization was proven effective, its performance is inadequate for practical applications, and further improvements are required. In this study, we only used six classes, including enamel and lesions of teeth. However, other lesions such as stains, dentin caries, and other areas such as the oral mucosa and gums were not considered. For the classification of dental lesions, multiclass classification and annotation are necessary. The solutions of the lighting weights obtained using the proposed method were unstable and differed from trial to trial. Multiple pieces of training were required under the different conditions of initial lighting weights to obtain improved performance.

The final goal of this study was to realize a system at a lower cost compared to more expensive systems and a longer imaging time. The proposed method has been developed, with the assumption of being replaced with other machine vision systems using spectral images; although, we used features obtained by simulating rendered images using spectral images to validate the proposed method. The rendering conditions, such as the distance and intensity of the light source, were not strictly considered, and upon its realization, the system’s successful operation could not be guaranteed. Therefore, the validation of the recognition performance of building an imaging system in reality, the estimation of measuring time, and the costs are required.

In addition, although the deep learning methods utilizing spectral imaging without lighting optimization have been developed, comparative verification of the proposed method to those applications is not shown in this study. Thus, the clarification of its accuracy is needed as compared to other methods such as [36,37,38,39]. Since the discrimination with the small patch image is inefficient for comparison to the methods, the application of the proposed method to the semantic segmentation task and the comparison to the recent studies, are required for validating the use of the proposed methods.

## 5. Conclusions

By optimizing the light-source spectrum, the color information of objects can be emphasized. Moreover, the performance of image recognition systems can be improved by using images with enhanced information as the input. In this study, we proposed a method for optimizing the light-source spectrum using deep learning and compared it with conventional methods. 

The proposed methods, NN-based and CNN-based optimization, yielded superior classification performance than when the D65 light source was used without light-source spectrum optimization, indicating the effectiveness of optimizing the light-source spectrum in NN-based image recognition. Compared with the conventional alternating optimization method, the proposed method improved the performance of the one-vs-rest classification in the F1-score. Notably, the effect of optimizing the light-source spectrum on the image recognition via neural networks was demonstrated in the limited problem of a one-vs-rest classification of small image patches.

The model used in this study was a CNN that performed a two-class classification using a small 5×5 image as the input; the scale of the model was small. To verify the practical use of the model, it should be trained with larger input images with more complex models and applied to various tasks other than the two-class classification, such as semantic segmentation.

## Figures and Tables

**Table 1:** Conditions of a classification problem.

Methods	Previous	Proposals	
	Alternating Optimization (Linear Support Vector Machine)	Neural Network (NN)	Convolutional Neural Network (CNN)
Input	1×1 pixel		5×5 image
Task	One-vs-rest, 2-class Classification		
Target	Class of pix		Class of the central pixel
Light source	1 24 light-emitting diodes (optimization), 2 D65 (Fixed, as reference)		
Cross Validation	five-Fold		
Initial lighting weights	Randomized vector in $\left[-1,\;1\right]$		

**Table 2:** The hyperparameters of NN and CNN for the grid search.

Parameters	NN-Based	CNN-Based
# of layers	$\left\{3,5,7\right\}$	
# of units	$\left\{10,15,20\right\}$	
Activation	{Rectified linear unit (ReLU), None}	
Conv2d	-	Size = 3 × 3, stride = 1, 10×3-channels
Output layer	Softmax	
Dropout	p=0.3	

**Table 3:** The specification of sub-lights.

Sub-Light	Peek Wavelength [nm]	
	Measured	Simulated
1	405	412
2	420	437
3	435	462
4	450	487
5	470	512
6	490	537
7	505	562
8	525	587
9	535	612
10	555	637
11	565	662
12	570	687
13	590	712
14	600	737
15	610	762
16	625	787
17	630	812
18	645	837
19	660	862
20	670	887
21	680	912
22	690	937
23	700	962
24	780	987

**Table 4:** Samples chosen for the one-vs-rest classification.

Class	Previous	Proposal	
	Alternating Optimization, 1×1	NN, 1×1	CNN,5×5
Enamel	11,836	11,836	11,836
Attrition and Erosion	2500	2500	2500
Calculus	1608	1608	1608
Initial Caries	792	792	792
Microfracture	900	900	900
Root	897	897	897

**Table 5:** The sample size for each class in the one-vs-rest classification.

Class	Sample Size	
	One	Rest
Enamel	11,836	6697
Attrition and Erosion	2500	16,033
Calculus	1608	16,925
Initial Caries	792	17,741
Microfracture	900	17,633
Root	897	17,636

## Data Availability

Publicly available datasets were analyzed in this study. This data can be found here: https://sites.uef.fi/spectral/odsi-db/ (accessed on 20 December 2022).
